# Density-dependent oxylipin production in natural diatom communities: possible implications for plankton dynamics

**DOI:** 10.1038/s41396-019-0518-5

**Published:** 2019-10-14

**Authors:** Ennio Russo, Giuliana d’Ippolito, Angelo Fontana, Diana Sarno, Domenico D’Alelio, Greta Busseni, Adrianna Ianora, Eric von Elert, Ylenia Carotenuto

**Affiliations:** 1 Stazione Zoologica Anton Dohrn, Department of Integrative Marine Ecology, Villa Comunale, 80121 Napoli, Italy; 2 Consiglio Nazionale delle Ricerche, Institute of Bio-molecular Chemistry, Via Campi Flegrei 34, 80078 Pozzuoli, Italy; 3 Aquatic Chemical Ecology Group, Institute for Zoology, Universität zu Köln, Zülpicher Straße 47b, D-50674 Köln, Germany

**Keywords:** Ecology, Ecology

## Abstract

Oxylipins are important signal transduction lipoxygenase-derived products of fatty acids that regulate a variety of physiological and pathological processes in plants and animals. In marine diatoms, these molecules can be highly bioactive, impacting zooplankton grazers, bacteria and other phytoplankton. However, the ultimate cause for oxylipin production in diatoms is still poorly understood, from an evolutionary perspective. Here we analysed production of particulate linear oxygenated fatty acids (LOFAs, previously named non-volatile oxylipins) from natural phytoplankton collected weekly for 1 year. We demonstrate for the first time that diatoms are the main LOFA producers in natural phytoplankton assemblages. Interestingly, LOFA-per-cell production decreased with increasing diatom density and was not due to major changes in diatom community composition. An inverse relation was confirmed at a global scale by analysing diatom lipoxygenase unigenes and metagenomes from *Tara* Oceans datasets. A network analysis suggested that different LOFAs could contribute to modulate co-variations of different diatom taxa. Overall, we offer new insights in diatom chemical ecology, possibly explaining the evolution of oxylipin synthesis in diatoms.

## Introduction

In marine systems, diatoms are one of the most important phytoplankton groups actively synthesizing oxylipins from membrane-bound polyunsaturated fatty acids upon activation by lipoxygenases (LOX) [1–9]. Diatoms mostly produce two major families of oxylipins, namely polyunsaturated aldehydes (PUAs) and non-volatile oxylipins (hereafter termed linear oxygenated fatty acids, LOFAs, because the concept of volatility in water systems is strongly misleading) [10].

Oxylipins have been reported from mono-specific diatom cultures and natural phytoplankton communities in the last two decades [11–19]. Laboratory evidence suggests that oxylipins can deter grazing [20–22], affect the growth of diatom-associated bacteria [23–25] and inhibit the growth of several phytoplankton taxa in a dose-dependent manner [26, 27]. These results suggest a role of oxylipins as defensive or allelopathic compounds to exclude predators or competitors as well as a role as info-chemicals synchronizing cell death of phytoplankton blooms [28–30]. The role of oxylipins in diatom chemical ecology is still debated and, in this perspective, field data can pave the way for hypothesis-driven laboratory setups. Nonetheless, only few field surveys have so far investigated oxylipin variations in time and space [18, 31–37].

We here report the first qualitative and quantitative characterization of diatom particulate LOFAs (i.e. LOFAs synthesized after inducing cell disruption) from natural phytoplankton communities at a weekly scale along one sampling year in the Gulf of Naples (GoN). Advanced methods for LOFA detection coupled with the simultaneous description of phytoplankton composition from the same water sample gave high resolution to our analysis. We demonstrate that oxylipin-per-cell production significantly decreases with increasing diatom density and validate this pattern at a global scale by exploring *Tara* Oceans data. A network analysis supports the view that oxylipins may contribute to modulate diatom–diatom interactions, either directly or indirectly. Our study sheds light on LOFA variations at sea in relation to phytoplankton abundance and composition and provides novel information on the role of these chemicals in natural diatom communities.

## Materials and methods

Samplings were taken weekly from January to December 2017 in the GoN, at the Long-Term Ecological-Research Station MareChiara (LTER-MC; 40°48.5′N, 14°15′E) [38, 39]. Environmental variables were measured as indicated in [40]. Water samples were collected at surface (0.5 m) with 10 L plastic buckets and pre-filtered onto a 200 µm mesh nylon net in order to remove large debris and meso-zooplankton. 250 mL were transferred into brown-glass bottles, fixed with formaldehyde (final concentration of ~1.6%) and stored at 4 °C in the dark. For LOFA analysis, a variable volume (0.5–2 L) of the same seawater was filtered onto Millipore polycarbonate filters (47 mm diameter, 2 µm mesh size, Merck KGaA, Darmstadt, Germany) using a multiple vacuum filtration three-place manifold apparatus (Merck KGaA, Darmstadt, Germany) mounting three 500 mL magnetic filter funnel beakers (VWR International S.r.l., Milan, Italy). Filters were immediately frozen in liquid nitrogen and stored at −80 °C.

Surface phytoplankton was identified and counted with a Zeiss Axiovert200 (Zeiss, Oberkochen, Germany) inverted microscope following the Utermöhl method [41]. Depending on cell concentration, subsamples of 3–50 mL were settled and cells were counted on one/two transects (each transect representing 1/30 of the chamber bottom) at ×400 magnification. Larger (>20 µm) and less abundant species were quantified in half of the chamber at lower magnification (×200). When possible, diatoms were identified to the species level. Most of the other taxa were clustered in major taxonomic groups. Abundance was expressed as cells/L. For biomass calculation, average bio-volumes available for the different species from LTER-MC data series were converted to carbon (ng-C/L) using formulas by [42].

For LOFA extraction, 1 mL of Milli-Q water was added to frozen filters. Samples were sonicated and left for 30 min at room temperature. Subsequent steps followed a standardized protocol for oxylipin extraction and quantification [6]. 16-Hydroxyhexadecanoic acid (1 µg) was added as internal standard. After extraction, samples were methylated with diazomethane, dried under nitrogen flow and finally re-dissolved in methanol before transferring them into a glass insert. Targeted LOFAs were quantified in ESI^+^ mode through Q Exactive Hybrid Quadrupole Orbitrap (Thermo Scientific, Waltham, USA) equipped with an Infinity 1290-HPLC-System (Agilent Technologies, Santa Clara, USA) mounting a reverse-phase Aquity-BEH-C18-column (1.7 µm, 2.1 × 50 mm; Waters, Milford, USA). Elution was set following methods described in [43]. LOFAs deriving from hexadecatrienoic (HTrA), eicosapentaenoic (EPA) and docosahexaenoic (DHA) fatty acids were targeted, because these molecules are known to constitute the bulk of oxylipin precursors in diatoms [6, 7, 14, 42]. LOFAs were quantified as follows: *ng*_(*x*)_ = (*a*_(*x*)_ × 1000)/*a*_(*s*)_, where “*x*” are oxylipin, “*S*” is the standard and “*a*” is the area of the peak in the chromatogram (Fig. S1). Absolute amount of each LOFA was normalized by the volume of water filtered and expressed as ng-LOFAs/L. LOFAs were also expressed as fg-LOFAs/diatom cell considering total diatom abundance in samples (diatom cells/L) [31].

### Data and network analyses

Each week, one sample replicate for oxylipin quantification was considered [44]. Monthly variations of LOFAs were estimated through a one-way analysis of similarities (ANOSIM) test (permutation *N* = 9999) based on Bray–Curtis dissimilarity. ANOSIM is a rank-based multivariate analysis allowing testing differences between two or more classes of objects [45]. A balanced design was produced considering group means as missing observations [46]. Similarity percentages (SIMPER) analysis allowed discerning main contributors of variations among classes of objects [47].

Relations between oxylipins and phytoplankton were explored through simple and multiple linear regression tests excluding observations available for the February 8 and June 20, because they fell outside the 95% distribution interval. Data were log-transformed to obtain linearity. To identify phytoplankton groups significantly driving LOFA production, a backward multiple linear regression was run considering diatoms, dinoflagellates, phytoflagellates and coccolithophores as predicting variables. Test assumptions were verified through the “gvlma” package implemented in R. Variance inflation factor (VIF) was calculated to exclude multicollinearity among predicting variables. As diatoms alone explained almost 90% of LOFA variation (see “Results” section), subsequent analyses only considered diatoms.

Simple linear regressions were conducted to test dependency of both LOFAs/L and LOFAs/diatom cell on diatom abundances and biomass. Linear models considering diatom abundance were refined through backward multiple linear regressions considering main diatom taxa as predicting variables. Here, rare species were clustered in the respective genus to reach a number of predicting variables (35 taxa) lower than the number of observations (*N* = 38). Assumptions of the tests were verified, but VIF was ignored, because the purpose here was to refine the general model [48].

To validate the predominant role of diatom density in driving LOFA-per-litre concentrations and LOFA-per-cell production, multiple linear regressions considering environmental variables and diatom density as possible predictors were performed. Environmental variables included temperature (°C), transmittance (%), fluorescence (RFU), oxygen-saturation (%), oxygen (mg/L), salinity (PSU), density (kg/m^3^) and chlorophyll-a (mg/m^3^) [40]. Variables showing VIF > 10 were removed from the model.

Freely available *Tara* Oceans datasets (http://tara-oceans.mio.osupytheas.fr/ocean-gene-atlas/) were inspected using as query the hidden Markov model (HMM) profile relative to LOX (LOX protein family, code number PFAM PF00305, *E*-values < 1^−10^). PFAM database is a large collection of protein families, each represented by multiple sequence alignment and HMMs [49]. LOX catalyse oxygenation of fatty acid precursors and are key factors in oxylipin synthesis processes [6]. From all the lipoxygenase transcripts detected, only unigenes annotated as “Bacillariophyta” within the MATOU database [50] were taken into account. A complete description of the sampling strategy and analytical protocols adopted for *Tara* Oceans expedition can be found in [51, 52]. Analyses involved only surface samples to allow closer comparison with our data collected in the GoN; all size classes available (ranging from 0.8 µm to 2000 µm) were considered in the analysis. LOX unigenes were normalized over transcript abundances of all the unigenes annotated as diatom products. Results were plotted in relation to the genomic abundances of the same diatom unigenes (i.e. a proxy for diatom abundance at the sampling location). Relation between normalized LOX unigenes and diatom metagenomes was inspected through a simple linear regression analysis.

To test if oxylipin-per-cell production (fg-LOFA/diatom cell) at LTER-MC was driven by changes in diatom community composition, three oxylipin concentration ranges were arbitrarily identified based on the observed results: “low”, “medium” and “high”, corresponding to 0–100, 100–300 and >300 fg/diatom cell, respectively. Each phytoplankton sample was clustered to the respective oxylipin concentration range. The list of diatom taxa was the same used for the multiple linear regressions, which allowed accounting for major changes in diatom community composition. Significant variations were tested through a one-way ANOSIM test (permutation *N* = 9999; Bray–Curtis index for raw data and Jaccard index for presence/absence data), balanced in replicate numbers as described before [46]. The ANOSIM test was supported by SIMPER analyses. Non-metric multidimensional scaling (nMDS) were produced to visualize results.

To better investigate the possible role of LOFAs as possible chemical factors modulating diatom–diatom links, co-variations of cell abundances were inspected, and data gathered therein were integrated by means of network analysis. As a preliminary step, consecutive weeks characterized by the persistence of the same water mass over time at LTER-MC were identified on the basis of salinity values [53]. Seven successive weeks (*N* = 7) were therefore selected in February–March, while 3 weeks (*N* = 3) were identified in each of 3 additional months (i.e. April, May and October). Salinity ranges in these periods were: 38 ± 0.06 PSU in February–March, 37.5 ± 0.12 PSU in April, 37.7 ± 0.05 PSU in May and 38.2 ± 0.01 PSU in October (Fig. S2).

Pairwise Spearman’s rank correlations were calculated among the abundances of diatom species observed in each period (i.e. February–March, April, May and October). Significant correlations (−0.7 ≥ ρ ≥ 0.7; *p* < 0.05) were taken into consideration and were subsequently used as input data for network analysis. Here, diatom taxa were nodes and positive/negative co-variations were edges.

Finally, mean concentrations of LOFAs expressed as fg/diatom cell in the four periods were calculated and considered on the basis of fatty acid precursors. hydroxy-hexadecatrienoic acid (HHTrE) and epoxy-hydroxy-hexadecadienoic acid (EHHDE) were clustered as HTrA-derived LOFAs; hydroxy-eicosapentaenoic acid (HEPE) and epoxy-hydroxy-eicosatetraenoic acid (EHETE) as EPA-derived LOFAs; hydroxy-docosahexaenoic acid and epoxy-hydroxy-docosapentaenoic acid (EHDPE) as DHA-derived LOFAs (Table S1). These mean concentrations allowed categorizing network edges on the basis of LOFA production (expressed as fg/diatom cell).

Concerning network analysis, the main effort focused on characterizing the overall organization of the interaction web, based on both network topology (i.e. degree of connection between nodes) and architecture (i.e. the differential clustering of connections around specific groups of nodes). To outline network architecture, network modularity (i.e. the presence of modules or groups of nodes more reciprocally interconnected) was estimated using the MORO application [54] implemented in Cytoscape 3.6 [55].

Node size was set proportional to degree (a measure of the number of edges that characterizes a node), calculated by Centiscape 2.2 implemented in Cytoscape 3.6. Edge colours mirrored the concentrations of LOFA classes (HTrA-derived, EPA-derived and DHA-derived LOFAs), thus describing species co-variations on the basis of LOFA-per-cell production.

To test whether distribution of LOFA production in module-1 (C16:3-derived LOFAs) and module-2 (C20:5-derived and C22:6-derived LOFAs) were random, null networks were constructed by random re-shuffling of mean LOFA productions among modules. *T*-tests were applied to verify significant variations in LOFA production from random values, assuming unequal variances when necessary (i.e. when *F*-test for variance dissimilarity was significant, *p* < 0.05).

Statistical analyses were performed in PAST 3.0 [56], R (version 3.5.0, packages “gvlma”, “car” and “ggplot2”) and Microsoft Excel. All tests were run considering two-tailed distributions. Network analysis and visualization were run in Cytoscape 3.6.

## Results

### Phytoplankton community abundance and composition

**Fig. 1:**
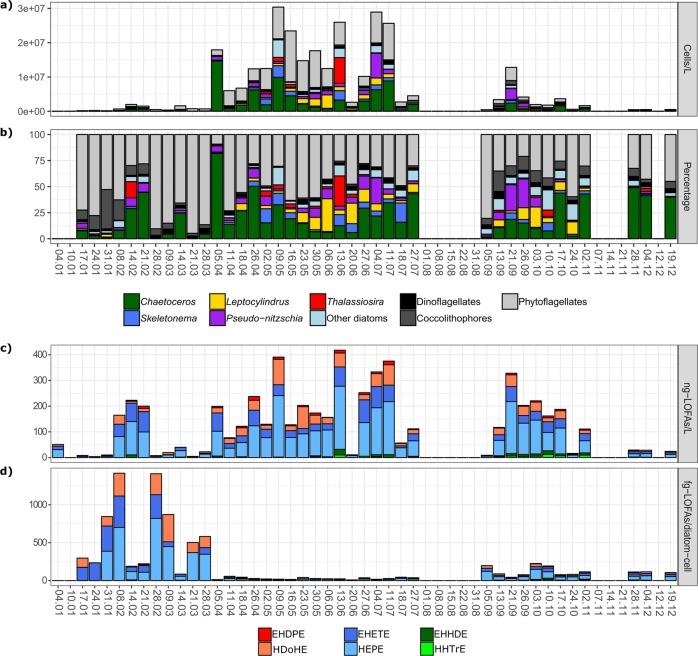
Weekly total phytoplankton abundance and composition at LTER-MC from January to December 2017 (**a** cell concentration; **b** relative percentage). The five most abundant diatom genera and the multispecies group “Other diatoms” are represented by coloured bars. The three other major taxonomic groups are represented by grey shading bars. Missing points indicate no sampling. **c**, **d** Linear oxygenated fatty acid (LOFA) quantification at LTER-MC in 2017 (**a** ng-LOFAs/L; **b** fg-LOFAs/diatom cell). Colours indicate fatty acid precursors of the respective oxylipin species: red = C22:6 derivatives, blue = C20:5 derivatives, green = C16:3 derivatives. Colour shadings indicate oxylipin species. Dark red: EHDPE epoxy-hydroxy-docosapentaenoic acid (C22:6); dark blue: EHETE epoxy-hydroxy-eicosatetraenoic acid (C20:5); dark green: EHHDE epoxy-hydroxyl-hexadecadienoic acid (C16:3); coral: HDoHE hydroxy-docosahexaenoic acid (C22:6); cyan: HEPE hydroxy-eicosapentaenoic acid (C20:5); green: HHTrE hydroxyl-hexadecatrienoic acid (C16:3). Missing points indicate no sampling. fg-LOFAs/diatom cell on January 4 are not shown because phytoplankton abundances are not available for this date

High phytoplankton abundance was observed from April to July, while low values were detected in March and November. Sudden fluctuations in cell density were frequently recorded (e.g. April 5 and June 20). Overall, diatoms and small phytoflagellates (mainly cells < 10 µm) dominated the phytoplankton community throughout the year (Fig. 1a, b). The former dominated phytoplankton assemblages during peaks observed in late spring, summer and September–October; the latter peaked in May–June, often constituting more than half of the total phytoplankton at minimum abundance values (mainly in January–March). Coccolithophores, mostly represented by *Emiliania huxleyi*, reached their highest abundances in September and October (up to 1.2 × 10^6^ cells/L), but their contribution was also relevant in winter (3–38%). Dinoflagellates reached their maximum density in June–July (up to 1 × 10^6^ cells/L), even though they scarcely contributed to the total phytoplankton abundance throughout the year (average 2.6 ± 1.4%).

In total, 58 diatom taxa were identified (Table S2). The five most abundant genera are shown in Fig. 1a, b. *Chaetoceros* was the most persistent genus along the year, dominating the phytoplankton community in spring (maximum 80% on April 5^th^) and autumn (usually around 50%). In winter, *Chaetoceros* was mostly represented by large colonial species (e.g. *C. affinis*, *C. curvisetus*, *C. protuberans* and *C. pseudocurvisetus*), while small single-cell species (mainly *C. tenuissimus* and *C. throndsenii*) occurred in the rest of the year. Undetermined *Chaetoceros* taxa (named *Chaetoceros* spp.) always occurred at considerable concentrations (mean value of 1.1 × 10^6^ cells/L). Other diatom genera showed occasional dominance: *Pseudo-nitzschia* (represented by *P. fraudulenta*, *P. galaxiae* and two species complexes, i.e. *P. delicatissima* and *P. pseudodelicatissima*), was present throughout the year and was mainly found from spring to autumn (maximum 7.2 × 10^6^ cells/L, on July 4^th^). The genus *Leptocylindrus* mainly occurred in late spring-summer and autumn. In the GoN, the genus *Leptocylindrus* includes *L. convexus* and three cryptic species, i.e. *L. danicus*, *L. hargravesii* and *L. aporus*, here grouped as *L*. cf. *danicus*. Occurrence of *L*. *convexus* was mainly limited to spring-summer months (maximum 1.9 × 10^6^ cells/L, on June 6^th^), while *L*. cf. *danicus* was found mostly all over the year (maximum 2 × 10^6^ cells/L, on June 6^th^). *Skeletonema pseudocostatum* was abundant in late spring-summer, and *S. tropicum* occurred in October/November. The genus *Thalassiosira* mainly occurred in summer and autumn. Blooms of small sized *Thalassiosira* (<10 µm) were observed in isolated weeks (maximum abundance of 7.5 × 10^6^ cells/L on June 13^th^). Rare diatom taxa (e.g. *Bacteriastrum* spp., *Dactyliosolen* spp., *Hemiaulus* spp., *Rhizosolenia* spp., *Thalassionema* spp.) were clustered together in the group of “Other diatoms”. Their contribution was particularly relevant on discrete dates in summer.

### Particulate LOFA concentration and composition

Six LOFAs including epoxy-alcohols and hydroxy acids originating from HTrA (HHTrE and EHHDE), EPA (HEPE and EHETE) and DHA (HDoHE and EHDPE) were targeted (Fig. 1c). Only few extracts were sufficiently concentrated to clearly determine molecular structures through UV absorbance or characteristic MS/MS fragmentation of epoxy-alcohols [6], then hindering inference on the type of LOX pathways from the position of hydroxy and epoxy groups along the alkyl chains.

Total LOFA-per-litre concentration varied widely throughout the year, ranging from a maximum of 417 ng/L (on June 13^th^) to a minimum of 3.6 ng/L (on January 24^th^; Fig. 1c). Concentrations briskly changed during the year, but three main periods of maximal abundance were identified: February, April–July and September–October (Fig. 1c). Hydroxy acids were the most abundant oxylipins; epoxy-alcohols were less concentrated (Table 1). The main fatty acid precursor of LOFAs was EPA, followed by DHA and HTrA. More specifically, HEPE was on average the most abundant LOFA (74.7 ± 68 ng/L), followed by EHETE (29.6 ± 27 ng/L) and HHTrE (3.1 ± 5 ng/L). HEPE, EHETE and HDoHE were the most persistent oxylipins: each of these molecules was always present except for two weeks. The one-way ANOSIM test revealed overall significant changes in oxylipin concentration and composition (Global-*R* = 0.25; *p* < 0.001), even if the *R* score was rather low and indicated similar oxylipin composition along the year. SIMPER analysis suggested that the HEPE and EHETE mainly drove variations, because they contributed for almost 75% of total dissimilarity.

**Table 1:** Linear oxygenated fatty acid (LOFA) concentrations expressed as ng/L and as fg/diatom cell observed in the sampling period (January–December 2017) at the Long-Term Ecological-Research Station MareChiara (LTER-MC)

LOFAs	Concentration		Range	
	ng/L	fg/diatom cell	ng/L	fg/diatom cell
EHDPE	3.82 ± 4.02	1.69 ± 2.51	0–14.92	0–9.97
HDoHE	22.56 ± 23.79	45.42 ± 86.32	0–99.02	0–359.66
EHETE	29.61 ± 26.61	55.05 ± 98.53	0–88.67	0–414.36
HEPE	**74.71** **±** **68.37**	**109.43** **±** **188.68**	0–**245.66**	0–**820.31**
HHTrE	1.96 ± 3.14	1.46 ± 3.12	0–12.17	0–14.49
EHHDE	3.11 ± 5.05	1.47 ± 3.46	0–22.04	0–16.78
**Total**	**135.95** **±** **118.5**	**214.51** **±** **346.47**		
Hydroxy acids	**33.08** **±** **51.64**	**52.10** **±** **126.86**	0–**340.21**	0–**1097.50**
Epoxy-alcohols	12.18 ± 19.98	19.40 ± 61.87	0–108.29	0–414.36
HTrA-derivatives	2.54 ± 0.81	1.46 ± 3.27	0–32.12	0–31.27
EPA-derivatives	**52.16** **±** **31.89**	**82.24** **±** **152.04**	3.26–**320.62**	6.12–**1133.58**
DHA-derivatives	13.19 ± 13.25	23.55 ± 64.54	0–107.68	0–359.66

Values are reported for each LOFA species, group (hydroxy acids and epoxy-alcohols) and fatty acid precursor (HTrA, EPA and DHA). Concentrations are given as mean ± SD, while ranges indicate the absolute minima and maxima. Highest values are highlighted in bold

*EHDPE* epoxy-hydroxy-docosapentaenoic acid (C22:6), *EHETE* epoxy-hydroxy-eicosatetraenoic acid (C20:5), *EHHDE* epoxy-hydroxyl-hexadecadienoic acid (C16:3), *HDoHE* hydroxy-docosahexaenoic acid (C22:6), *HEPE* hydroxy-eicosapentaenoic acid (C20:5), *HHTrE* hydroxyl-hexadecatrienoic acid (C16:3)

LOFA production (fg/diatom cell) showed a different annual pattern (Figs. 1d and S3). The highest concentrations were detected in winter, when each diatom cell was estimated to synthesize 1410–1417 fg-LOFAs. In February, other peaks of lower intensity were observed. A potential to synthesize more than 100 fg-LOFAs/diatom cell was detected in autumn (Fig. 1d). The annual average cellular production of hydroxy acids was higher than that for epoxy-alcohols (Table 1). On average, HEPE was the most abundant oxylipin produced by each diatom cell (109.4 ± 188.7 fg/diatom cell), followed by EHETE. The average cellular production of EHDPE, HHTrE and HEpHTrE were sensibly lower (Table 1; Fig. S3).

### Diatom–LOFA interactions

**Fig. 2:**
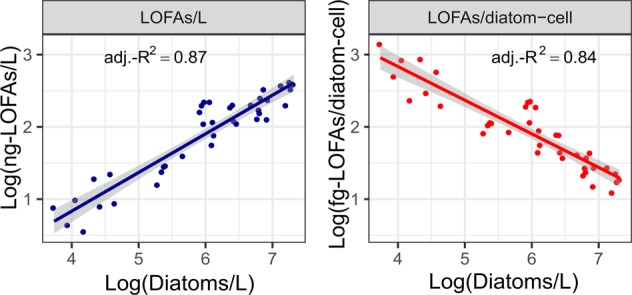
Linear regressions between diatom abundances and linear oxygenated fatty acid (LOFA) concentrations. **a** Blue dots and line indicate the relationship between ng-LOFAs/L and diatoms/L. **b** Red dots and line indicate the relationship between fg-LOFAs/diatom cell and diatoms/L. In both **a** and **b** adjusted-*R*^2^ (adj.-*R*^2^) values are shown. Grey shadings indicate the 95% confidence interval of the linear regressions. Log-transformed data are shown

Backward multiple linear regression analysis identified the main phytoplankton groups responsible for production of the targeted oxylipins. Diatoms and coccolithophores offered the best prediction (multiple-adjusted-*R*^2^ = 0.92, *F* = 217, d.f. = 2 and 35, *p* < 0.001; *N* = 38); contribution of dinoflagellates and phytoflagellates was not significant (*p* > 0.05). To determine which group between diatoms and coccolithophores best predicted LOFA-per-litre variations, single linear regression analyses were performed, considering these two groups separately. Diatoms (*y* = 0.52x + 2.7; adjusted-*R*^2^ = 0.87; *F* = 253.6; d.f. = 36; *p* < 0.001; *N* = 38; Fig. 2) better related to ng-LOFAs/L concentrations than coccolithophores (*y* = 0.93x + 6.2; adjusted-*R*^2^ = 0.61; *F* = 59.44; d.f. = 36; *p* < 0.001; *N* = 38). Considering also the low density of coccolithophores at LTER-MC in 2017, further analyses on LOFA-per-litre concentrations were performed only in relation to diatoms. The linear regression between diatoms and ng-LOFAs/L was refined through a multiple linear regression analysis: those diatom taxa that did not significantly contribute to oxylipin production were excluded, and the model resulted in a multiple-adjusted-*R*^2^ = 0.98 (*F* = 93.61, d.f. = 27 and 10, *p* < 0.001; *N* = 38).

A significant inverse regression was observed (*y* = −0.46x + 10.75; *R*^2^ = 0.84; *F* = 192.8; d.f. = 36; *p* < 0.001; *N* = 38; Fig. 2) between diatom abundance and LOFA-per-cell production (fg/diatom cell). In this case, a multiple linear regression test slightly improved the general model (multiple-adjusted-*R*^2^ = 0.89, *F* = 33.39, d.f. = 9 and 28, *p* < 0.001; *N* = 38). Weak inverse linear regressions were observed, when calculations were performed considering phytoflagellates (*y* = −0.31x + 3.66; adjusted-*R*^2^ = 0.18, *F* = 7.98, d.f. = 36, *p* < 0.01, *N* = 38), dinoflagellates (*y* = −0.26x + 4.32; adjusted-*R*^2^ = 0.21, *F* = 9.46, d.f. = 36, *p* < 0.01, *N* = 38) and coccolithophores (*y* = −0.05x + 3.16; adjusted-*R*^2^ = 0.004, *F* = 0.13, d.f. = 36, *p* > 0.05, *N* = 38).

To verify whether the regressions with LOFAs were valid also when considering diatom biomass, total diatom carbon (ng-C/L) was plotted against LOFA concentration (ng-LOFAs/L) and LOFA production (fg-LOFAs/ng-diatom-C). Regressions were significant, but the adjusted-*R*^2^ were considerably lower than those obtained with diatom abundance (Fig. S4). The predominant role of diatoms in driving LOFA variations was further supported by the non-significant effects (*p* > 0.05) of the environmental variables considered in the multiple linear models. In particular, gradients in both ng-LOFAs/L and fg-LOFAs/diatom cell were significantly explained by diatoms and chl-a, but not by the other environmental variables (Table S3).

**Fig. 3:**
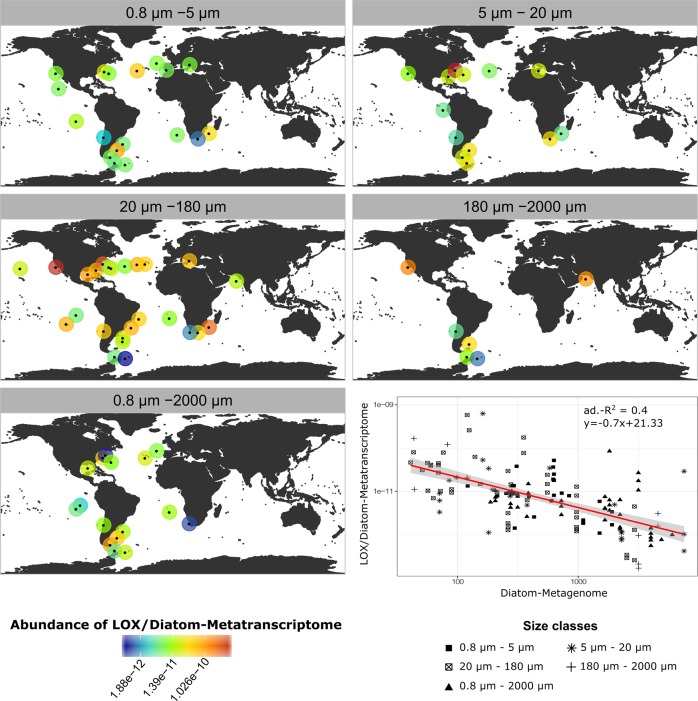
Global maps showing distribution of *Tara* Oceans sampling stations where LOX unigenes from Bacillariophyta were found. Circle colours indicate concentration of LOX unigenes normalized by the abundance of diatom transcripts. Each map refers to the respective size class range (µm). Plot in the bottom shows the linear regression between diatom lipoxygenase (LOX) unigenes normalized by diatom unigenes (metatranscriptome) and total genomic abundances of diatom unigenes (metagenome). Data are in log-scale. Size classes indicate size range (µm) of samples. Adjusted-*R*^2^ (adj.-*R*^2^) value and equation of the linear regression are shown. Grey shading along the regression curve (red line) indicates the 95% confidence interval of the regression

Linear regression analysis considering *Tara* Oceans data revealed a significant inverse relation between the expression of LOX unigenes depending on genomic abundances of diatom unigenes (*y* = −0.7x + 21.33; *R*^2^ = 0.4; *F* = 96; d.f. = 144; *p* < 0.001; *N* = 146; Fig. 3). This analysis shows how the expression of LOX unigenes normalized by total diatom transcripts varies depending on genomic diatom sequences, which was considered as a proxy of diatom abundance.

**Fig. 4:**
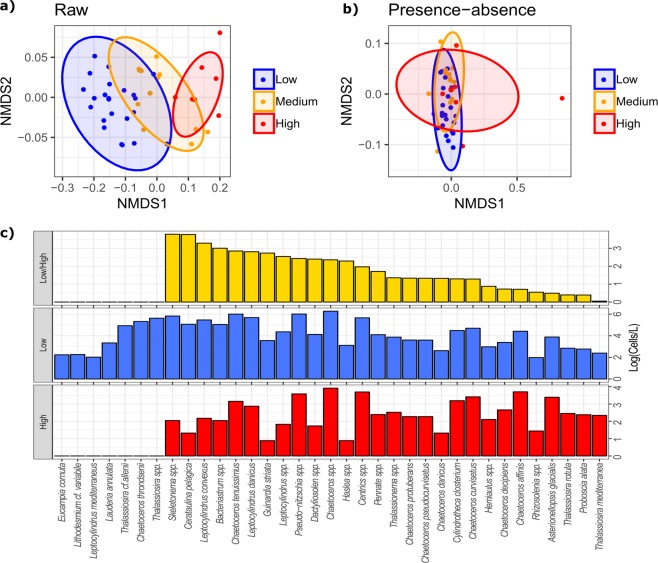
**a** nMDS representing three oxylipin concentration groups on the basis of their similarities in diatom community composition considering raw abundance data (stress = 0.07). **b** nMDS representing three oxylipin concentration groups on the basis of their similarities in diatom community composition considering presence/absence data (stress = 0.2). 95% ellipses are shown; ellipse colours indicate observations characterized by different LOFA-per-cell productions: blue = “low” (0–100 fg/diatom cell), orange = “medium” (100–300 fg/diatom cell), red = “high” (>300 fg/diatom cell). **c** SIMPER analysis results based on raw diatom abundances. Diatom taxa are listed on abscissas; cellular concentrations are shown on ordinates. Diatom concentrations were log-transformed for clarity. Red bars indicate abundance of the respective diatom species contributing to community of “high” (>300) fg-LOFAs/diatom cell. Blue bars indicate abundance of the respective diatom species contributing to community of “low” (0–100) fg-LOFAs/diatom cell. Yellow bars indicate the ratio between diatom densities in the “low” and the “high” condition and are ordered from the highest to the lowest values. Higher ratios indicate higher differences in cellular concentrations of the respective species in the “low” and the “high” observations

The balanced ANOSIM test inspecting whether changes in fg-LOFAs/diatom cell were driven by variations in diatom community composition highlighted highly significant differences (Global-*R* = 0.75, *p* < 0.001). Diatom composition characterizing the group “high” was mostly separated from the group “low” (*R* = 0.96, *p* < 0.001). The group “medium” showed intermediate values (*R* = 0.51 vs “low”; *R* = 0.76 vs “high”). nMDS was effective, showing a stress = 0.07 (Fig. 4a). To verify if these differences were driven by diatom abundance rather than composition, the ANOSIM test was repeated on presence–absence data, which allowed accounting only for differences in taxonomic composition (Fig. 4b). The test revealed low differences (Global-*R* = 0.22, *p* < 0.001), and group similarity was much higher than the one observed for raw data (*R* = 0.26, *p* < 0.001 “low” vs “high”; *R* = 0.25, *p* < 0.001 “medium” vs “low”; *R* = 0.19, *p* < 0.001 “medium” vs “high”). SIMPER analysis indicated that only few diatom taxa did not contribute to the diatom composition of the “high” concentration group in comparison to the “low” group and that *Thalassiosira mediterranea*, *Proboscia alata*, *Thalassiosira rotula*, *Asterionellopsis glacialis* and *Rhizosolenia* spp. showed the lowest concentration differences in the “low” and the “high” oxylipin-per-cell production groups (Fig. 4c).

### Network analysis

**Fig. 5:**
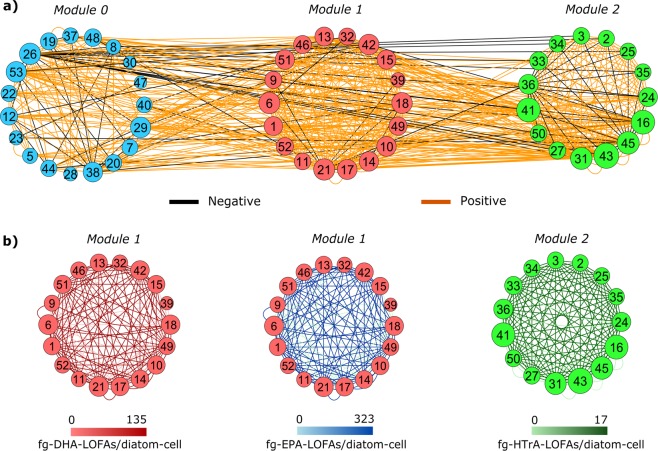
**a** Diatom links in the sampling weeks selected for network analysis. Numbers indicate the node ID, the size of the nodes is based on the degree of each node. Colours of the nodes vary based on modularity (blue = module-0; red = module-1; green = module-2). **b** Single network modules showing edge categorization on the basis of EPA-derived (blue edges), DHA-derived (red edges) and HTrA-derived (green edges) oxylipins (fg-LOFAs/diatom cell). Black edges indicate negative links. Coloured bars indicate variations in edge colours depending on oxylipin concentration

Network analysis clustered 52 out of 53 nodes, i.e. diatom taxa (node 4 was not included in the network because of no links, Table 2), in three distinct modules characterized by a different number of interacting species (Fig. 5a). Positive links dominated in the network (average positive degree = 18.4, average negative degree = 2.8).

**Table 2:** Diatom species, genera and morphological groups (Centrics and Pennates) identified at the Long-Term Ecological-Research Station MareChiara (LTER-MC) in February–March, April, May and October and used for network analysis

Diatom taxa	Node Id	Module	Diatom taxa	Node Id	Module
*Asterionellopsis glacialis*	1	**1**	*Hemiaulus hauckii*	28	**0**
*Bacteriastrum furcatum*	2	**2**	*Hemiaulus sinensis*	29	**0**
*Bacteriastrum jadranum*	3	**2**	*Lauderia annulata*	30	**0**
*Bacteriastrum parallelum*	4	**/**	*Leptocylindrus convexus*	31	**2**
*Cylindrotheca closterium*	5	**0**	*Leptocylindrus* cf. *danicus*	32	**1**
Centrics	6	**1**	*Leptocylindrus* spp.	33	**2**
*Chaetoceros affinis*	7	**0**	*Leptocylindrus mediterraneus*	34	**2**
*Chaetoceros anastomosans*	8	**0**	*Lithodesmium variabile*	35	**2**
*Chaetoceros curvisetus*	9	**1**	*Proboscia alata*	36	**2**
*Chaetoceros danicus*	10	**1**	Pennates	37	**0**
*Chaetoceros decipiens*	11	**1**	*Pseudo-nitzschia delicatissima*	38	**0**
*Chaetoceros diversus*	12	**0**	*Pseudo-nitzschia fraudulenta*	39	**1**
*Chaetoceros peruvianus*	13	**1**	*Pseudo-nitzschia galaxiae*	40	**0**
*Chaetoceros protuberans*	14	**1**	*Pseudo-nitzschia multistriata*	41	**2**
*Chaetoceros pseudocurvisetus*	15	**1**	*Pseudo-nitzschia pseudodelicatissima*	42	**1**
*Chaetoceros simplex*	16	**2**	*Pseudo-nitzschia* spp.	43	**2**
*Chaetoceros socialis*	17	**1**	*Rhizosolenia* spp.	44	**0**
*Chaetoceros* spp.	18	**1**	*Skeletonema pseudocostatum*	45	**2**
*Chaetoceros tenuissimus*	19	**0**	*Skeletonema* spp.	46	**1**
*Chaetoceros throndsenii*	20	**0**	*Skeletonema tropicum*	47	**0**
*Cerataulina pelagica*	21	**1**	*Thalassiosira* cf. *allenii*	48	**0**
*Dactyliosolen blavyanus*	22	**0**	*Thalassionema frauenfeldii*	49	**1**
*Dactyliosolen fragilissimus*	23	**0**	*Thalassiosira mediterranea*	50	**2**
*Dactyliosolen phuketensis*	24	**2**	*Thalassionema nitzschoides*	51	**1**
*Eucampia cornuta*	25	**2**	*Thalassiosira rotula*	52	**1**
*Guinardia striata*	26	**0**	*Thalassiosira* spp.	53	**0**
*Haslea* spp.	27	**2**			

Each taxonomic unit represents a node of the network (node Id is provided). The respective module of each species is indicated by numbers in bold (0, 1, 2)

The node 43 (i.e. *Pseudo-nitzschia* spp.) showed the highest degree in the network (=47). Intra-module negative edges were mostly observed in module-2 and module-0, while inter-module negative edges were related to node 26 (i.e. *Guinardia striata*), located in module-0.

Categorization of edges on the basis of fg-LOFAs/diatom cell (Table S1) highlighted that maximal densities of HTrA-derived LOFAs mostly characterized intra-module connections among nodes in module-2 (Fig. 5b). In contrast, the highest production of EPA- and DHA-derived oxylipins best described intra-module connections in module-1 (Fig. 5b).

*T*-tests were all significant (*p* < 0.001), suggesting non-random distribution of oxylipin concentrations among modules (*t* = −4.92, d.f. = 258, *N* = 130 for EPA-derived LOFAs in module-1; *t* = −4.49, d.f. = 258, *N* = 130 for DHA-derived LOFAs in module-1; *t* = −9.76, d.f. = 218, *N* = 110 for HTrA-derived LOFAs in module-2; Fig. S5).

## Discussion

Qualitative and quantitative characterization of particulate LOFAs from our data confirm that HTrA and EPA represent relevant oxylipin precursors in diatoms [1, 3, 5, 7, 36], but also suggest that the relative contribution of DHA at sea may be higher than previously thought [14]. In fact, previous information is based on a rather limited number of species, which were either absent (e.g. *Skeletonema marinoi*) or poorly abundant (e.g. *Thalassiosira rotula*) in the GoN during this study. Higher concentrations of hydroxy acids than epoxy-alcohols observed in our survey were described also in the Northern Adriatic Sea [36] and might indicate a common over-expression of reductases in natural diatom communities [7]. Analyses of mono-specific diatom cultures have revealed a wide variety of LOX pathways in the lineage even if the distribution of these enzymes is rather species-specific. Unfortunately, the inability to generate MS fragmentation of the epoxy-alcohol hinders sound consideration on the occurrence of different LOX pathways in the natural assemblage of the GoN.

Particulate oxylipin-per-litre concentration reported here range between earlier reports in the Atlantic Ocean [18], the Strait of Gibraltar [33] and the Northern Adriatic Sea [32]. As a general pattern, oxylipin-per-litre concentrations are known to vary widely (from pg/L to µg/L) depending on the study area, which suggests that oxylipin-per-litre concentrations depend on the phytoplankton abundance (with the highest concentrations in the hypereutrophic Northern Adriatic Sea, the lowest in the Atlantic Ocean) [18, 32]. Our regression analyses support this interpretation (Fig. 2) and provide the first evidence that the selected oxylipins analysed from natural phytoplankton communities derive mostly from diatoms. In this perspective, higher slope of the regression indicates a higher potential of natural diatom communities to produce LOFAs. Different slopes of this linear equation in different marine systems might prove to be very suitable to estimate oxylipin synthesis potential under in situ conditions. In contrast to oxylipin-per-litre concentrations, the magnitude of oxylipin-per-cell production (fg/diatom cell) seems not to vary with system productivity [18, 21, 33, 37].

Oxylipin production (fg/cell) detected in other field surveys seems in line with our interpretation that cellular oxylipin production is reduced as diatom abundances increase [31]. The same trend was recently reported by [56], who demonstrated an inverse relation between particulate PUAs/*chl* and *chl* (a proxy for phytoplankton biomass). We also detected a significant inverse relation between fg-LOFAs/diatom-C and ng-diatom-C/L, but variations in oxylipin production in our study were much better explained by diatom density.

The evidence that oxylipin-per-cell production is inversely related to diatom concentration is also valid at the global scale, as demonstrated by the simple linear regression analysis performed on *Tara* Oceans datasets. The latter analysis is based on the expression of LOX unigenes, which has been shown to well relate to oxylipin synthesis in diatoms [57]. This suggests that variations in oxylipin-per-cell production depend on physiological responses of diatoms at the transcription level with respect to LOX expression. Remarkably, this evidence has wide evolutionary, biological and ecological implications, because dependence of oxylipin synthesis potential on diatom density points at a major role of LOFAs as chemical signals. That is, higher oxylipin synthesis potentials at minimal diatom abundances may be driven by the necessity of diatoms to communicate with each other with stronger signals, when they are highly dispersed in the water medium. At high cell densities, instead, cell communication is guaranteed by lower oxylipin synthesis potential. In this way, cells reduce physiological costs of LOX expression and oxylipin synthesis, simultaneously maintaining information exchange.

Depending on reciprocal interconnections among diatom taxa, network analysis resulted in three modules, where interactions among diatom taxa were described by a different set of LOFAs. Maximal production of EPA- and DHA-derived LOFAs (expressed as fg/diatom cell) best described positive interactions of diatom taxa clustered in module-1. By contrast, HTrA-derived LOFAs mostly coincided with the positive interactions among diatom taxa in module-2 (Fig. 5b). Maximal production of these three oxylipin classes were highly dissimilar (Table S1) and their distribution across module-1 and module-2 was not stochastic (Fig. S5). This pattern suggests that HTrA-derived oxylipins could constitute a much more species-specific chemical signal for cellular communication among diatoms. In agreement with this hypothesis, HTrA-derived oxylipins, including octadienal, have been so far reported only in few diatom species, such as *Thalassiosira rotula* and *Skeletonema marinoi*. Instead, synthesis of LOFAs from EPA and DHA fatty acids could be privileged when diatoms need to produce a stronger signal, at lower cellular densities.

In our network, positive interactions dominate over the negative ones (Fig. 5a). However, we cannot distinguish among direct and indirect relationships. Direct positive and negative relations can be respectively mediated by either a higher stress resistance induced by exposure to sub-lethal oxylipin doses (*sensu* Vardi et al.) [29] or a cellular growth inhibition (*sensu* Casotti et al.) [27]. Indirect positive and negative relationships, instead, could be mediated by the effects of oxylipins on consumers such as micro-zooplankton [21] and/or meso-zooplankton [22].

**Fig. 6:**
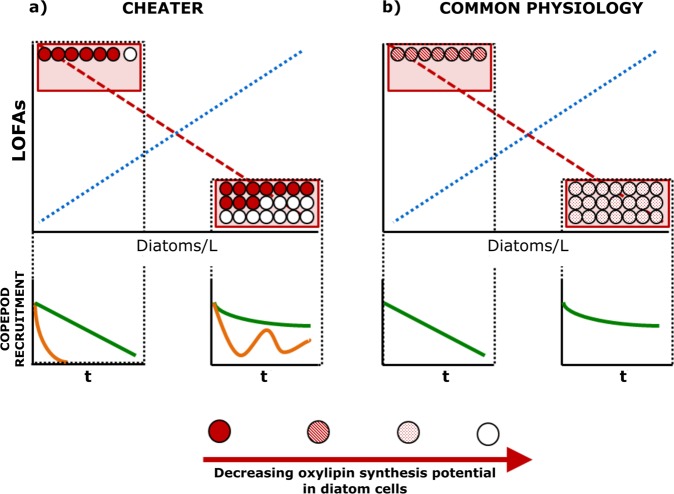
Conceptual model synthesizing two possible dynamics describing variations in linear oxygenated fatty acid (LOFA) synthesis potential along diatom density gradient. Circles indicate diatom cells and fill gradient indicates the oxylipin synthesis potential (red is the highest, white is the lowest). Dotted blue lines show the positive relation between ng-LOFAs/L and diatom cells/L; dashed red lines show the inverse relation between fg-LOFAs/diatom cell and diatom cells/L. **a** “Cheater” dynamic: some diatom cells can reduce oxylipin synthesis depending on the abundance of diatom cells with constantly high oxylipin synthesis potentials. At low diatom densities, diatom community is dominated by cells with high oxylipin synthesis potentials. In this condition, low ng-LOFAs/L are detected because of the low abundance of diatoms showing high oxylipin synthesis potentials. However, high fg-LOFAs/diatom cell are measured because community is dominated by cells with high synthesis potentials. At high diatom densities, both cheaters and oxylipin producers increase, thus more ng-LOFAs/L are detected, but lower fg-LOFAs/diatom cell are measured. After grazing on diatom cells at low and high abundances, copepod grazer recruitment can be hypothesized to vary depending on selective (orange lines) or unselective (green lines) feeding strategies. **b** “Common physiology” dynamic: all diatom cells share the same physiological adaptation and synthesize the same amount of molecules. At low diatom concentrations, higher oxylipin synthesis potentials are shown by each diatom cell. At high diatom densities, lower oxylipin synthesis potentials are shown by each diatom cell. Amount of ng-LOFAs/L and fg-LOFAs/diatom cell does not vary in comparison to the “cheater scenario”. Copepod grazer recruitment after feeding on diatom cells at low and high abundances is different because of different ingestion of harmful molecules, but it does not vary depending on selective or unselective feeding strategies

Importantly, our results indicate that different cellular oxylipin production in the GoN is not associated with major changes in diatom community composition, as demonstrated by the ANOSIM and SIMPER tests. This suggests that similar diatom communities can modulate oxylipin synthesis depending on diatom cell abundance. We do not exclude that different diatom species may show higher tendency to synthesize a particular set of oxylipin species, but the purpose of the analyses that we presented was to provide sound evidence about the primary role of diatom abundance rather than composition in driving variations in cellular oxylipin synthesis potential. Unfortunately, it remains unclear whether all diatom cells are capable of adjusting their oxylipin production over time. In this perspective, two main scenarios can be hypothesized (Fig. 6, b). 1) A “cheater” scenario: some diatom species always produce oxylipins, while others suppress the LOX enzymatic machinery involved in oxylipin synthesis, thereby reducing energy consumption and cheating on oxylipin producers, because these cheaters still take advantage of the information mediated by the chemical signal (Fig. 6a). 2) A “common physiology” scenario: all diatom species share the same physiology and are capable of changing oxylipin production depending on the abundance of diatom cells (Fig. 6b).

Each scenario has wide implications for the fitness of copepod grazers. When in the cheater scenario grazers ingest diatoms occurring at low abundances (cells/L; Fig. 6a), they have a high probability of ingesting a high amount of oxylipins, because the diatom community is dominated by cells with a high oxylipin synthesis potential. Therefore, grazer fitness can briskly decline along time after selective or unselective feeding. When, instead, feeding on diatoms occurs at high abundances, the probability of ingesting high amounts of oxylipins is reduced, because cheaters are relatively more abundant in the community. Therefore, copepod fitness after selective feeding can be oscillatory because of alternated ingestion of cheaters and cells showing high oxylipin synthesis potential. After unselective feeding, instead, copepod fitness can slowly decline along time because of mean low oxylipin ingestion.

When grazers ingest diatoms occurring at low abundances (cells/L) in the “common physiology” scenario (Fig. 6b), a high oxylipin uptake will occur independently of the diatom cell ingested. Thus, grazer fitness would progressively decrease along time irrespective of selective or unselective feeding strategies. Instead, when feeding occurs on diatoms at high abundances in this scenario, the amount of oxylipins ingested will be lower, because all diatom cells downregulate LOX pathways. In this case, grazer fitness can be supposed to resemble the one proposed in the “cheater” scenario at high diatom concentrations, independently of selective or unselective feeding.

Overall, our data put forward for the first time the predominant role of diatoms in synthesizing LOFAs in natural phytoplankton communities. Evidence provided at the local and the global scale suggests a major role of oxylipins, in our case LOFAs, as chemical signals which can potentially contribute to plankton community structuring. On this ground, we propose that the oxylipin synthesis machinery in marine diatoms could have originally selected in response to a necessity of diatom cells to communicate with each other, but afterwards they have assumed another function as mechanism of chemical defence. Understanding how variations in oxylipin production by diatom cells could impact natural plankton communities is challenging. The network approach in general, as we have presented here, and our results and conceptual models in particular can help decipher the importance of chemicals for plankton dynamics in marine systems.

## Supplementary information

Supplementary material
