# Supplementary material for: Density-dependent oxylipin production in natural diatom communities: possible implications for plankton dynamics
Source: ISME J. 2019 Oct 14;14(1):164–77. doi: 10.1038/s41396-019-0518-5 (PMC6908693; doi:10.1038/s41396-019-0518-5)
Supplement: Supplementary file 1 — Supplementary material [file 41396_2019_518_MOESM1_ESM.docx]

**SUPPLEMENTARY**


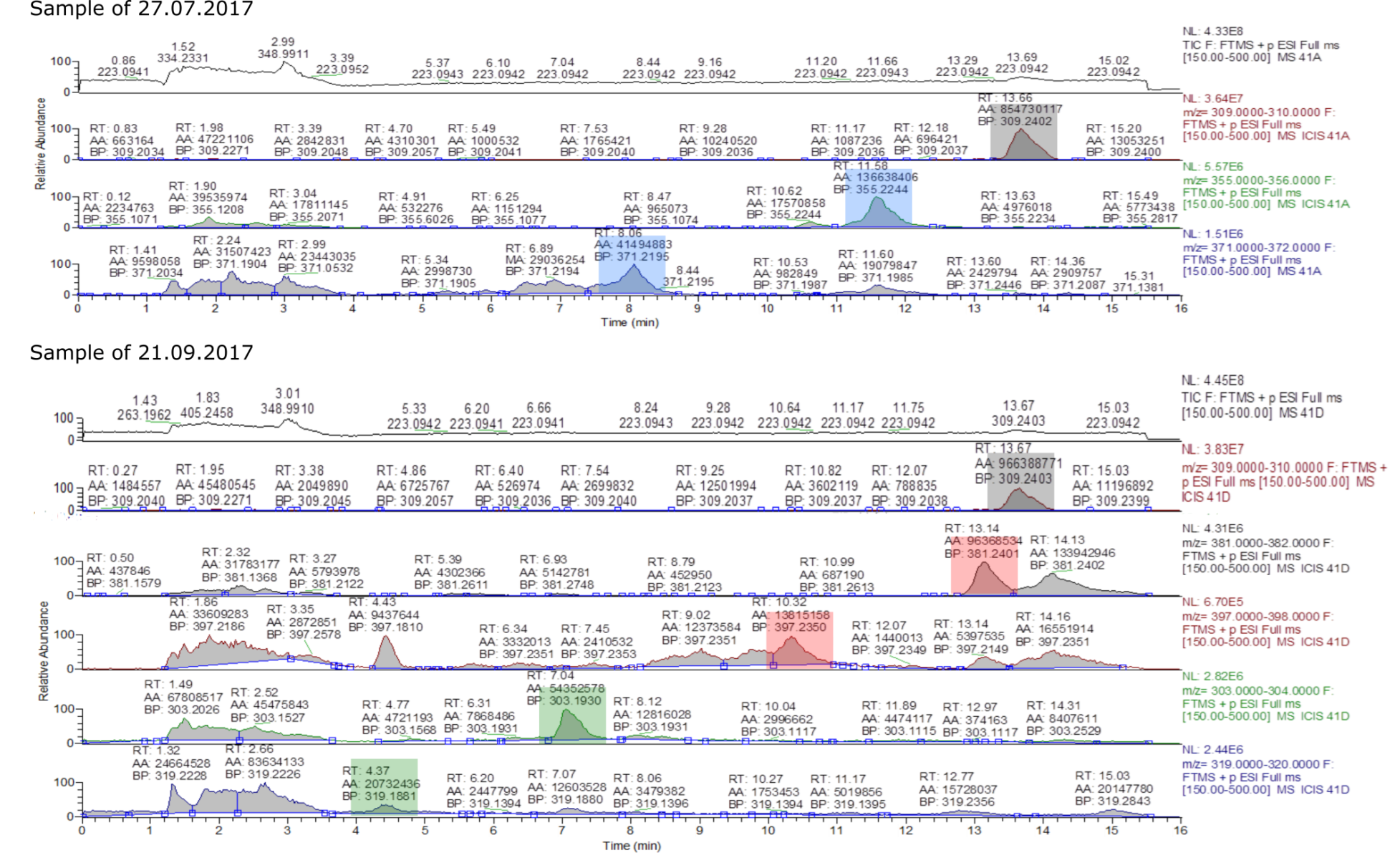


Figure S1. Chromatograms of extracts obtained from phytoplankton samples of 27.07.2017 and 21.09.2017 after oxylipin extraction. Coloured boxes highlight the compound of interest, discerned depending on mass (M+Na^+^ m/z) and retention time. M+Na^+^ m/z: 309=standard (grey); 355=HEPE (blue); 371=EHETE (blue); 381=HDoHE (red); 397=EHDPE (red); 303=HHTrE (green); 319=EHHDE (green).


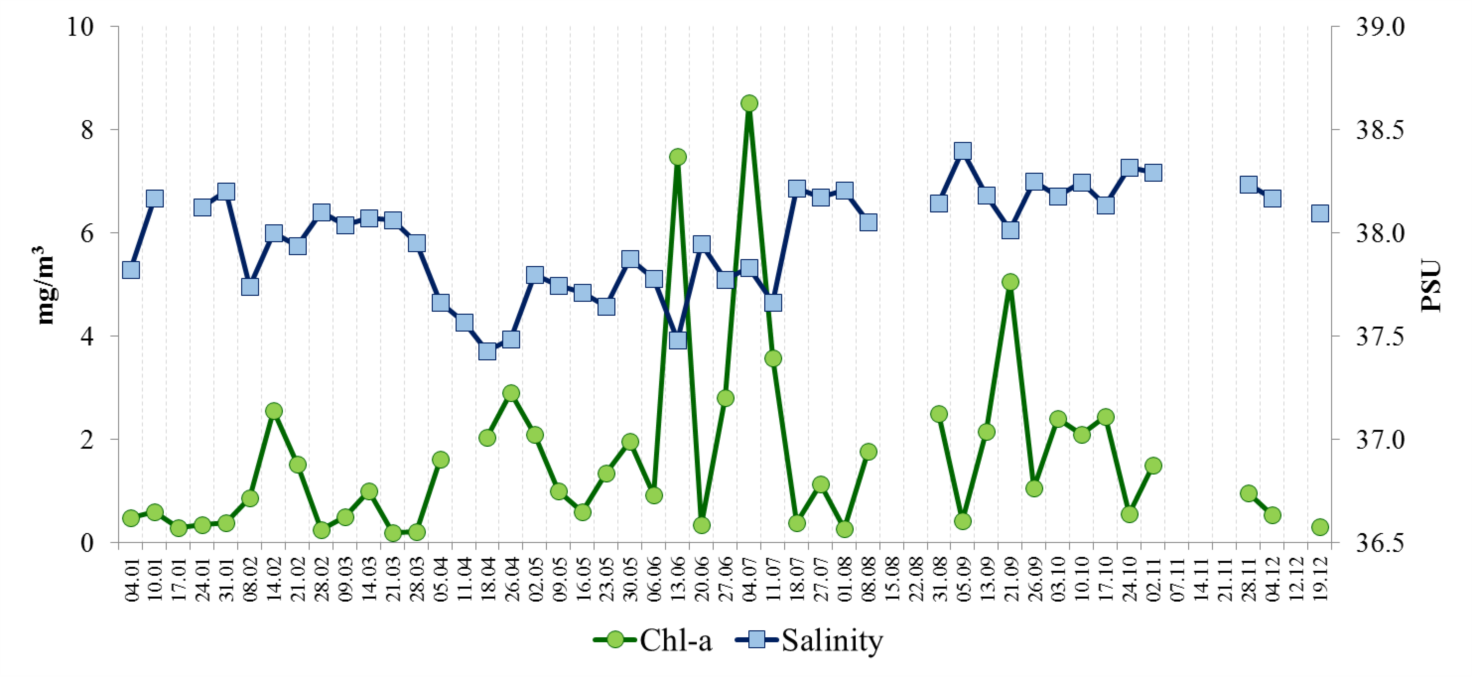


Figure S2. Chlorophyll-a (mg/m^3^) and salinity (PSU) oscillations measured at LTER-MC from January 2017 to December 2017.


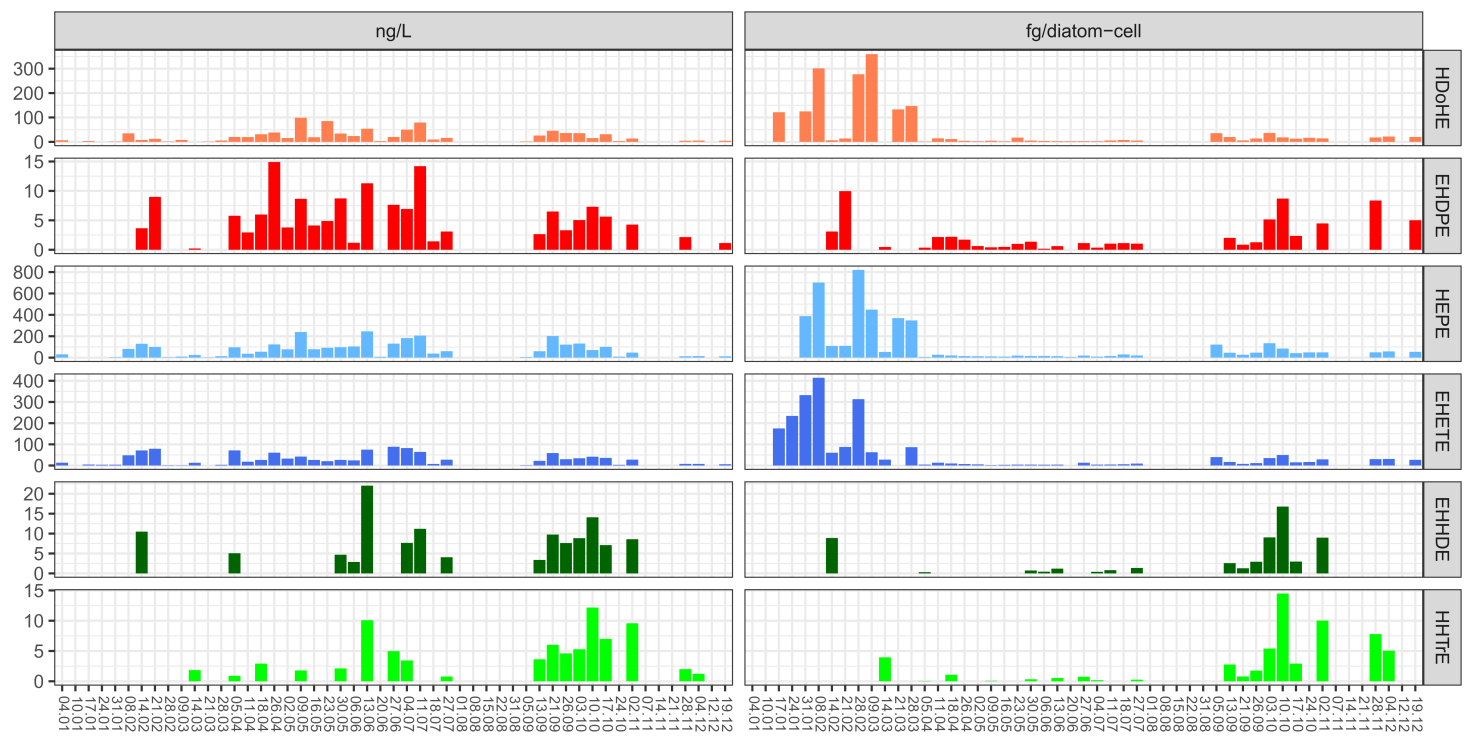


Figure S3. Linear oxygenated fatty acid (LOFA) quantification at LTER-MC in 2017. a) ng-LOFAs/L; b) fg-LOFAs/diatom cell. Colours indicate fatty acid precursors of the respective oxylipin species: red = C22:6 derivatives, blue = C20:5 derivatives, green = C16:3 derivatives. Colour shadings indicate oxylipin species. Dark red: EHDPE = Epoxy-Hydroxy-Docosapentaenoic-acid (C22:6); dark blue: EHETE=Epoxy-Hydroxy-Eicosatetraenoic-acid (C20:5); dark green: EHHDE=Epoxy-Hydroxyl-Hexadecadienoic-acid (C16:3); coral: HDoHE=Hydroxy-Docosahexaenoic-acid (C22:6); cyan: HEPE=Hydroxy-Eicosapentaenoic-acid (C20:5); green: HHTrE=Hydroxyl-Hexadecatrienoic-acid (C16:3). Missing points indicate no sampling. fg-LOFAs/diatom-cell on 4^th^ January are not shown because phytoplankton abundances are not available for this date.


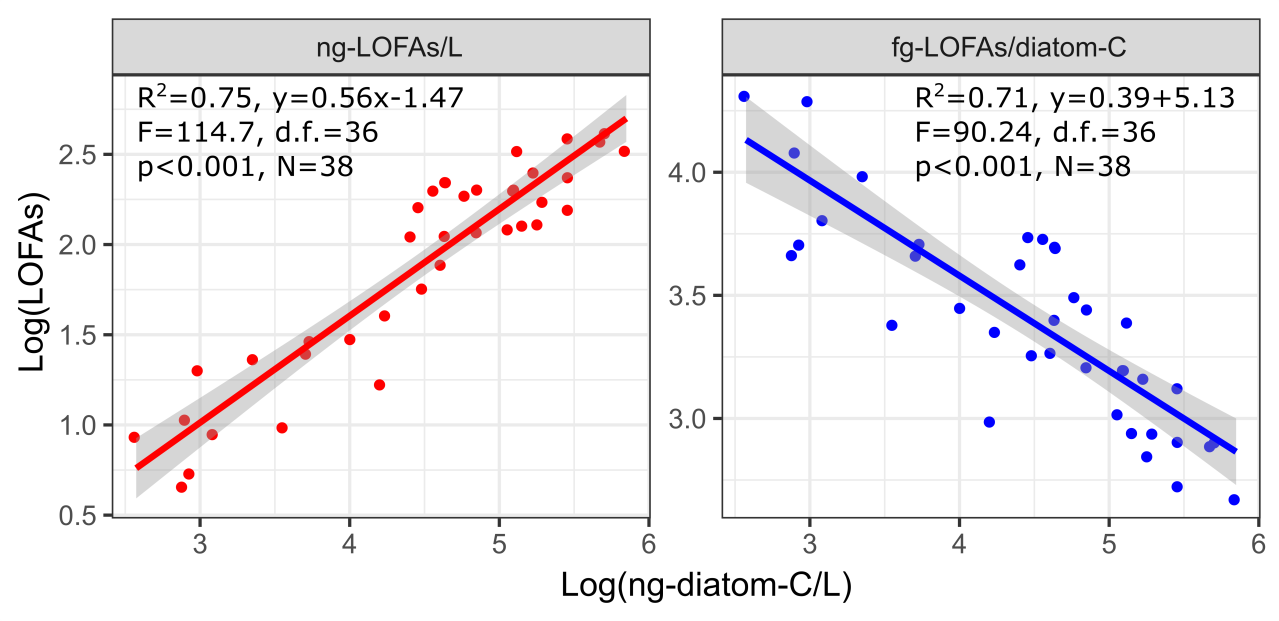


Figure S4. Linear regressions between LOFA concentration (ng/L) and cellular production (fg/diatom-C) and diatom-carbon-per-litre concentrations (ng-C/L). Adjusted-R^2^ and regression results are reported. Grey shading indicates the 95% confidence interval of the regression curve. Data were log-transformed.


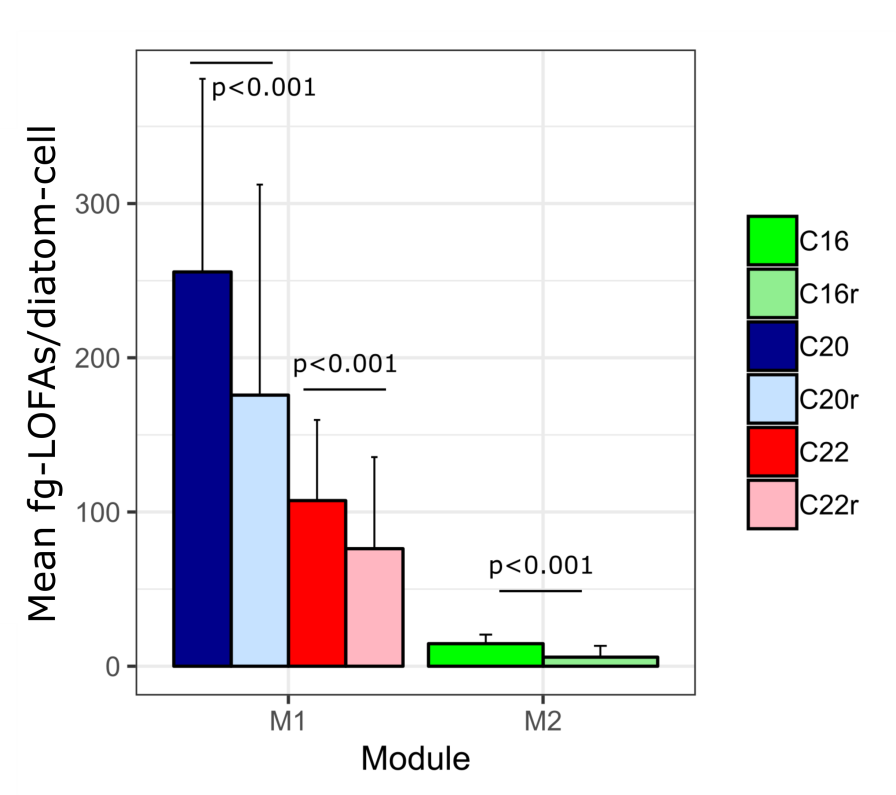


Figure S5. T-test results comparing concentrations of fg-LOFAs/diatom-cell in original (dark coloured C16, C20 and C22 bars) and randomized (light coloured C16r, C20r and C22r bars) network modules. P-value for each t-test is reported. Bars indicate average values ± SE.

Table S1. Mean (±SD) LOFA concentrations (fg/diatom-cell) calculated in the respective period and used as input data for network analyses. The highest concentrations of each oxylipin precursor are highlighted in bold.

|  | Months | | | |
| --- | --- | --- | --- | --- |
| Fatty acid-derived LOFA | *February-March* | *April* | *May* | *October* |
| *HTrA-derived* | 1.83 (±1.3) | 0.48 (±0.31) | 0.03 (±0.03) | **17.20 (±7.45)** |
| *EPA-derived* | **323.42 (±100.85)** | 13.93 (±4.44) | 13.24 (±2.89) | 95.64 (±28.2) |
| *DHA-derived* | **135.85 (±52.77)** | 7.14 (±3.52) | 8.78 (±4.87) | 28.05 (±7.59) |

Table S2. List of the 58 diatom taxa identified and of the mean bio-volumes estimated at LTER-MC. Mean carbon concentration for each species is also reported.

| **Species Name** | **Mean biovolume (µm^3^)** | **Mean carbon (ng-C/L)** |
| --- | --- | --- |
| *Asterionellopsis glacialis* | 534.44 | 46.92 |
| *Bacteriastrum furcatum* | 1564.32 | 112.10 |
| *Bacteriastrum jadranum* | 1357.03 | 99.89 |
| *Bacteriastrum parallelum* | 141.83 | 16.00 |
| *Bacteriastrum* spp. | 6859.12 | 279.74 |
| Centrics | 938.88 | 63.51 |
| *Cerataulina pelagica* | 6894.82 | 281.02 |
| *Chaetoceros affinis* | 2009.65 | 137.35 |
| *Chaetoceros anastomosans* | 889.97 | 70.95 |
| *Chaetoceros brevis* | 1782.99 | 124.65 |
| *Chaetoceros curvisetus* | 797.96 | 64.94 |
| *Chaetoceros dadayi* | 1319.34 | 97.64 |
| *Chaetoceros danicus* | 1962.02 | 134.70 |
| *Chaetoceros decipiens* | 3753.76 | 164.48 |
| *Chaetoceros diversus* | 217.91 | 22.66 |
| *Chaetoceros minimus* | 33.68 | 4.99 |
| *Chaetoceros peruvianus* | 2802.93 | 179.89 |
| *Chaetoceros protuberans* | 2391.46 | 158.16 |
| *Chaetoceros pseudocurvisetus* | 2286.26 | 152.49 |
| *Chaetoceros simplex* | 154.52 | 17.15 |
| *Chaetoceros socialis* | 197.10 | 20.89 |
| *Chaetoceros* spp. | 62.14 | 8.19 |
| *Chaetoceros tenuissimus* | 25.70 | 4.00 |
| *Chaetoceros throndsenii* | 22.16 | 3.55 |
| *Cylindrotheca closterium* | 116.02 | 13.59 |
| *Dactyliosolen blavyanus* | 33173.52 | 1121.55 |
| *Dactyliosolen fragilissimus* | 4182.33 | 180.91 |
| *Dactyliosolen phuketensis* | 2431.41 | 160.30 |
| *Eucampia cornuta* | 2310.41 | 153.80 |
| *Guinardia striata* | 22891.57 | 808.86 |
| *Haslea* spp. | 2415.13 | 159.32 |
| *Hemiaulus hauckii* | 7848.65 | 315.00 |
| *Hemiaulus sinensis* | 5102.44 | 215.55 |
| *Lauderia annulata* | 13513.76 | 508.41 |
| *Leptocylindrus convexus* | 700.00 | 58.39 |
| *Leptocylindrus danicus* | 700.00 | 58.39 |
| *Leptocylindrus mediterraneus* | 819.48 | 66.36 |
| *Leptocylindrus* spp. | 739.83 | 58.39 |
| *Lithodesmium* cf. *variabile* | 6590.29 | 270.06 |
| Pennates | 2479.78 | 117.04 |
| *Proboscia alata* | 9443.62 | 370.76 |
| *Pseudo-nitzschia delicatissima* | 105.20 | 12.56 |
| *Pseudo-nitzschia fraudulenta* | 993.65 | 77.58 |
| *Pseudo-nitzschia galaxiae* | 65.82 | 8.58 |
| *Pseudo-nitzschia multistriata* | 205.74 | 21.63 |
| *Pseudo-nitzschia pseudodelicatissima* | 184.17 | 19.77 |
| *Pseudo-nitzschia* spp. | 115.53 | 13.55 |
| *Rhizosolenia* spp. | 25444.37 | 887.82 |
| *Skeletonema menzelii* | 56.24 | 7.56 |
| *Skeletonema pseudocostatum* | 182.82 | 19.66 |
| *Skeletonema* spp. | 182.82 | 19.66 |
| *Skeletonema tropicum* | 993.21 | 77.55 |
| *Thalassionema frauenfeldii* | 1671.10 | 118.26 |
| *Thalassionema nitzschioides* | 508.08 | 45.03 |
| *Thalassiosira cf.allenii* | 192.40 | 20.49 |
| *Thalassiosira mediterranea* | 725.99 | 60.15 |
| *Thalassiosira rotula* | 8061.10 | 322.50 |
| *Thalassiosira* spp. | 327.40 | 31.53 |

Table S3. Multiple linear regressions testing the combined effects of environmental variables and diatoms on LOFA/L and LOFA/cell variations. The environmental variables were selected after removing factors showing Variance Inflation Factor (VIF)>10. After backward removal of the non-significant (p>0.05) variables from the model, only chl-a and diatoms resulted the significant predictors of the oxylipin concentrations.

| **Model** | **LOFAs~Trasmittance+Oxygen+Salinity+Density+Chl.a+Diatoms** | | | |
| --- | --- | --- | --- | --- |
| **Dependent variable** | ng-LOFAs/L | | fg-LOFAs/diatom-cell | |
|  | t | p | t | p |
| *Chl.a* | 3.023 | 0.0048 | 2.855 | 0.0074 |
| *Diatoms* | 9.398 | 7.49^-11^ | -12.283 | 7.42^-14^ |
|  | Adj.-R^2^=0.89, F=144.5, d.f.=2 and 33, p<0.001; N=38 | | Adj.-R^2^=0.87, F=113.1, d.f.=2 and 33, p<0.001; N=38 | |
